# Further investigations of the W-test for pairwise epistasis testing

**DOI:** 10.12688/wellcomeopenres.11926.1

**Published:** 2017-07-21

**Authors:** Richard Howey, Heather J. Cordell

**Affiliations:** 1 Institute of Genetic Medicine, Newcastle University, Newcastle upon Tyne, NE1 3BZ, UK

**Keywords:** Interactions, epistasis, GWAS, quality control, contingency table

## Abstract

**Background:** In a recent paper, a novel W-test for pairwise epistasis testing was proposed that appeared, in computer simulations, to have higher power than competing alternatives. Application to genome-wide bipolar data detected significant epistasis between SNPs in genes of relevant biological function. Network analysis indicated that the implicated genes formed two separate interaction networks, each containing genes highly related to autism and neurodegenerative disorders.

**Methods:** Here we investigate further the properties and performance of the W-test via theoretical evaluation, computer simulations and application to real data.

**Results:** We demonstrate that, for common variants, the W-test is closely related to several existing tests of association allowing for interaction, including logistic regression on 8 degrees of freedom, although logistic regression can show inflated type I error for low minor allele frequencies,  whereas the W-test shows good/conservative type I error control. Although in some situations the W-test can show higher power, logistic regression is not limited to tests on 8 degrees of freedom but can instead be tailored to impose greater structure on the assumed alternative hypothesis, offering a power advantage when the imposed structure matches the true structure.

**Conclusions: **The W-test is a potentially useful method for testing for association - without necessarily implying interaction - between genetic variants disease, particularly when one or more of the genetic variants are rare. For common variants, the advantages of the W-test are less clear, and, indeed, there are situations where existing methods perform better. In our investigations, we further uncover a number of problems with the practical implementation and application of the W-test (to bipolar disorder) previously described, apparently due to inadequate use of standard data quality-control procedures. This observation leads us to urge caution in interpretation of the previously-presented results, most of which we consider are highly likely to be artefacts.

## Introduction

In a paper recently published in the journal *Nucleic Acids Research*, Wang and colleagues ^1^ proposed a novel *W*-test for pairwise epistasis testing. The thrust of the proposed method was to compare the distributions of the *k* observed genotype combinations at *L* = 2 diallelic genetic loci such as single nucleotide polymorphisms (SNPs), between cases and controls (see Table 1). In general, for *L* = 2 loci, the number of possible categories *k* = 9, although this number can be lower if any cells are empty in cases and/or controls. The comparison of genotype distributions between cases and controls is achieved by constructing a cell-specific measure for each of the observed genotype categories, corresponding to the normalized log odds ratio for that category:

$$\log\frac{{\hat{p}}_{1i}/(1-{\hat{p}}_{1i})}{{\hat{p}}_{0i}/(1-{\hat{p}}_{0i})}/SE_{i}$$

where $\hat{p}$_1 *i*_ = *n*_1 *i*_ / *N*_1_, $\hat{p}$_0 *i*_ = *n*_0 *i*_ / *N*_0_ and

$$SE_{i}=\sqrt{\frac{1}{n_{0i}}+\frac{1}{n_{1i}}+\frac{1}{N_{0}-n_{0i}}+\frac{1}{N_{1}-n_{1i}}}$$

( *i* = 1, . . . *k*). These cell-specific quantities are then combined to construct a scaled *χ* ^2^ test statistic whose value and (possibly non-integer) degrees of freedom (df) can be calculated as a function of two parameters ( *h* and *f*) that are estimated using a bootstrapping approach. Although in principle applicable to other orders of SNP combinations, including single SNPs (where *k* = 3) and combinations of more than two SNPs (where *k* = 3 *^L^*), the main focus of the published paper ^1^ and accompanying software was on the pairwise test.

**Table 1.** 2 × *k* contingency table for *L* = 2 loci (resulting in *k* = 9 genotype combinations). The two possible alleles at each locus (which for SNPs will corrrespond to bases A, C, G or T) are labelled as 1 and 2.

SNP1 *×*SNP2 genotype (allele1/allele2)										
	1/1 *×*1/1	1/1 *×*1/2	1/1 *×*2/2	1/2 *×*1/1	1/2 *×*1/2	1/2 *×*2/2	2/2 *×*1/1	2/2 *×*1/2	2/2 *×*2/2	Total
Cases	*n*_11_	*n*_12_	*n*_13_	*n*_14_	*n*_15_	*n*_16_	*n*_17_	*n*_18_	*n*_19_	*N*_1_
Controls	*n*_01_	*n*_02_	*n*_03_	*n*_04_	*n*_05_	*n*_06_	*n*_07_	*n*_08_	*n*_09_	*N*_0_

Analysis of 2 *× k* contingency tables, such as Table 1, in order to detect association between the row and column variables, is a classical problem in statistics that has had a long history of investigation in the statistical and epidemiological literatures. The usual treatment depends on whether one wishes to condition on the row margins (here, the number of cases and controls – as would be natural in a case/control study), on the column margins (the ‘exposure’ variables – as would be natural in a clinical trial or a cohort study), or both ^2, 3^. Conditioning on the number of cases and controls leads to considering the cell counts as coming from two independent multinomial distributions. Conditioning on the column margins leads to considering the cell counts as coming from *k* independent binomial distributions. Conditioning on both row and column margins leads to a hypergeometric distribution for the cell counts.

Tests of association within each of the above formulations depend on whether one wishes to use an exact or an asymptotic test ^2, 3^. Interestingly, all formulations result in a *χ* ^2^ test statistic on *k −* 1 = 8 df (provided all cells are represented in the data), reflecting the fact that there are *k −* 1 additional independent parameters to be estimated under the alternative hypothesis (where the row and column variables are allowed to be associated) compared to the null hypothesis (where there is no association between the row and column variables). This contrasts with the *W*-test proposed by Wang *et al.* ^1^, in which *k* = 9 non-independent (log odds ratio) quantities are combined, resulting in the necessity for a scaled *χ* ^2^ test statistic (with parameters *h* and *f* estimated using bootstrapping) in order to account for the non-independence between the *k* = 9 normalized log odds ratios.

Arguably the most natural way to analyse data from a 2 *×* 9 contingency table is to perform a standard Pearson’s *χ* ^2^ test ^4^ on 8 df, testing the independence of the column variable (here, genotype) and the row variable (here, case/control status). We note that both Pearson’s *χ* ^2^ test and the *W*-test actually test for *association* (between genotype category and phenotype) rather than testing specifically for *epistasis* or *statistical interaction* ^5– 9^ between the genotypes at the two loci in relation to phenotype. Unfortunately, depending on the software implementation used, Pearson’s *χ* ^2^ test can fail to produce a test statistic for sparse data (i.e. cells with low or zero genotype frequencies), and continuity corrections ^10^ have only been developed for 2 *×*2 and 2 *×*1 contingency tables. Wang *et al.* ^1^ point out that one important advantage of their proposed *W*-test is its adaptive ability to cope with sparse data, through the data-dependent bootstrap estimation of the scaling factor *h* and the degrees of freedom parameter *f*.

The precise details of Wang *et al.*’s bootstrap procedure are not fully delineated in their manuscript, but perusal of both the manuscript and the R code provided indicates that the bootstrap involves using a default of *B* = 200 bootstrap replicates, each of which uses genotype data from *N _B_* = min (1000, *N*) randomly chosen individuals and *P _B_* = min (1000, *P*) randomly chosen pairs of SNPs (where *N* is the total number of individuals and *P* the total number of pairs of SNPs in the data set under study), along with phenotype data (case/control status) that are resampled under the null hypothesis (i.e. independent of genotype). Thus, in order to implement the proposed bootstrap procedure, one needs real data from a reasonable number of ‘other’ pairs of SNPs, which are used as ‘surrogates’ (to estimate the distributional properties of the test) for every ‘test’ pair of SNPs. Thus the bootstrap procedure is data-adaptive in the sense that real GWAS data (at a number of pairs of SNPs – possibly but not necessarily including the test pair) from the current data set are used to estimate the non-independence (covariances) between the *k* = 9 normalized log odds ratios for each test pair of SNPs. However, the rationale for using *P _B_* ‘other’ pairs of SNPs as surrogates for each test pair of SNPs – and for then keeping the resulting estimates of *h* and *f* constant for each test pair of SNPs – is not discussed by Wang *et al.* ^1^. Standard statistical theory dictates that the asymptotic variances/covariances of the parameter estimates $\hat{p}$_*ji*_ (where *j* = 1 refers to cases and *j* = 0 to controls, and considering the cell counts in cases and controls as coming from two independent multinomial distributions) can be calculated ^3^ as [ **Diag**( *π*) - *ππ ^T^*]/ *N _j_*, where *π* is the vector of true underlying multinomial probability parameters *π ^T^* = ( *p*_*j*1_, *p*_*j*2_, . . . , *p*_*j*9_), whose maximum likelihood estimates are $\hat{p}$_*ji*_ = *n _ji_ /N _i_*, and **Diag**( *π*) is the diagonal matrix:

$$\left[\begin{array}{l} \begin{array}{ccccccccc} p_{j1} & 0 & 0 & 0 & 0 & 0 & 0 & 0 & 0\\ 0 & p_{j2} & 0 & 0 & 0 & 0 & 0 & 0 & 0\\ 0 & 0 & p_{j3} & 0 & 0 & 0 & 0 & 0 & 0\\ 0 & 0 & 0 & p_{j4} & 0 & 0 & 0 & 0 & 0\\ 0 & 0 & 0 & 0 & p_{j5} & 0 & 0 & 0 & 0\\ 0 & 0 & 0 & 0 & 0 & p_{j6} & 0 & 0 & 0\\ 0 & 0 & 0 & 0 & 0 & 0 & p_{j7} & 0 & 0\\ 0 & 0 & 0 & 0 & 0 & 0 & 0 & p_{j8} & 0\\ 0 & 0 & 0 & 0 & 0 & 0 & 0 & 0 & p_{j9} \end{array} \end{array}\right]$$

This suggests that the covariances of the normalized log odds ratios, which are functions of the estimates $\hat{p}$_*ji*_, could perhaps be better estimated on the basis of the observed data at the test pair of SNPs alone, possibly through use of a bootstrap. This would presumably result in a data-adaptive approach that adapts to the properties of the specific SNP pair under test, rather than to the properties of all (or a sample of) other SNP pairs in the data set, many of which may have quite different properties (e.g. different minor allele frequencies (MAFs)) from the SNP pair under test.

In the GWAS literature, use of the full set of (or a sample of) observed test statistics as, in some sense, ‘surrogates’ for the test statistic at any specific SNP (in order to estimate the distributional properties of the test) is not unusual. Devlin and Roeder ^11^ showed that, in the presence of population stratification, the genome-wide distribution of *χ* ^2^ test statistics on 1 df (when testing for allelic association between genotype and phenotype) is inflated by a constant multiplicative factor *λ*. Devlin and Roeder therefore proposed estimating this ‘genomic control’ factor *λ* on the basis of a sample of observed test statistics, and producing a set of adjusted test statistics by dividing each of the observed test statistics by *λ*. This suggestion is closely related to the now popular approach ^12^ of using quantile-quantile (Q-Q) plots to investigate whether there is any evidence for population stratification (or indeed some other phenomenon that causes departure from the expected genome-wide distribution of test statistics) in a GWAS. If the resulting plot of the observed test statistics versus their expected values shows sufficient departure from the line of equality, then each observed test statistic can be adjusted by dividing it by an estimate of *λ*. The bootstrap procedure proposed by Wang *et al.* ^1^ for estimating *h* and *f* would therefore appear to fall into this general framework. Thus, while theoretical arguments would suggest that a data-adaptive estimation of *h* and *f* might most naturally depend only on the data at the SNP pair currently under test, the use of data from a large number of other pairs of SNPs to estimate the relevant distribution can be perhaps motivated by comparison to Devlin and Roeder’s genomic control procedure. Moreover, given that the implementation of the proposed bootstrap procedure actually generates 9 different values of *h* and *f* (dependent on the number of observed genotype categories, *k*), one could argue that the only ‘other’ SNPs that contribute to determining the distribution for any test pair of SNPs are those that have similar properties to the test SNP, at least in terms of the sparsity of the observed genotype table.

In their paper, Wang *et al.* compared their proposed *W*-test to Pearson’s *χ* ^2^ test, as well as to Multifactor Dimensionality Reduction ^13^ and to an unspecified (possibly a linear allelic) logistic regression test. Another method, not – as far as we are aware – considered by Wang *et al.*, for comparing the distributions of *k* = 9 (or possibly less) genotype categories between two groups (cases and controls) would be to carry out a saturated logistic regression test on 8 (or possibly less) df, i.e. comparing (via a likelihood ratio test) a model:

                                logit( *p*)  = *α* + *β*_1_ *I*( *x*_1_ = 1) + *β*_2_ *I*( *x*_1_ = 2)

                                                   +γ _1_ *I*( *x*_2_ = 1) + γ _2_ *I*( *x*_2_ = 2)

                                                   + *i*_11_ *I*( *x*_1_ = 1) *I*( *x*_2_ = 1) + *i*_12_ *I*( *x*_1_ = 1) *I*( *x*_2_ = 2)

                                                   + *i*_21_ *I*( *x*_1_ = 2) *I*( *x*_2_ = 1) + *i*_22_ *I*( *x*_1_ = 2) *I*( *x*_2_ = 2)

with a null model logit( *p*) = *α*, where *p* denotes the probability of an observation being a case (rather than a control), *x*_1_ and *x*_2_ denote the genotypes (coded 0, 1, 2) at locus 1 and 2 respectively, and *I* represents an indicator function. The parameters ( *β*_1_, *β*_2_, *γ*_1_, *γ*_2_) correspond to the main effects of locus 1 and 2 respectively, and the four *i _st_* parameters correspond to statistical interaction effects (on the logit scale). The 9 parameters ( *α*, *β _s_*, *γ _t_*, *i _st_*) (where *s* and *t* each take values 1 or 2) are essentially reparameterisations of the 9 independent parameters *δ _uv_* obtained when modelling the log odds of disease given genotype (where *u* and *v* each take values 0, 1 or 2 according to genotype at locus 1 and 2) as:

                                                          logit( *p*) = log( *p*/(1 – *p*)) = *δ _uv_*,

– in other words, allowing the log odds (and thus the probability) of disease to take 9 different values according to the genotype category to which an individual belongs. Any completely missing genotype categories in cases and/or controls result in one or more parameters being dropped from the model, thus the method automatically adapts to the sparsity of the observed data (albeit in a different way from Wang *et al.*’s data-adaptive procedure). This logistic regression formulation emphasizes the fact that the 8 df test actually tests for association (which could correspond to main effects, interaction effects, or both), rather than testing for statistical interaction *per se*.

This ‘prospective’ logistic regression model (modelling the log odds of outcome or phenotype, given exposure or genotype) is in contrast to Wang *et al.*’s ‘retrospective’ model, which models (the ratio of) the log odds for genotype given phenotype. The prospective model is most natural in the context of cohort studies or clinical trials, but is arguably less natural in the context of case control studies, where subjects are ascertained based on their phenotype (case or control status). However, it has been shown ^14^ that valid estimates of the parameters of interest ( *β _s_*, *γ _t_*, *i _st_*) – which correspond to ratios of the log odds for phenotype (i.e. the log odds of being a case rather than a control) at different levels of the exposure variables – are achieved when this prospective model is applied to retrospectively ascertained case/control data. This convenient property has resulted in the enduring popularity of logistic regression as the standard method of choice in the epidemiological literature for analysing case/control data.

## Methods and Results

### Application of *W*-test to real and simulated example data sets

To compare the newly-proposed *W*-test with other, more standard, analysis options, we applied the *W*-test and three alternative methods (Pearson’s *χ* ^2^ test using two alternative implementations, and logistic regression on 8 df (denoted LR8)) to two different data sets. The first data set was distributed with the *W*-test software (R version) developed by Wang *et al.* ^1^. This example data set consists of 50 SNPs (resulting in 1225 SNP pairs) genotyped in 1000 individuals, and presumably corresponds to a single replicate of the simulated data (simulated under an interaction model, using real genotype data) described by Wang *et al.* ^1^. The second data set was a simulated data set that we constructed ourselves using real genotype data from Wellcome Trust Case Control Consortium 2 (WTCCC2) controls ^15^, with phenotype (case/control status) simulated under the null hypothesis of no difference in genotype distribution between cases and controls. Specifically, we selected 1000 female founder individuals and randomly assigned 500 as cases and 500 as controls. Real genotype data were selected at 50 SNPs for these individuals by first LD pruning using the PLINK ^16^ command "–indep-pairwise 50 5 0.5" and then the first and last five SNPs from chromosomes 1 to 5 were extracted, giving 50 SNPs in total.

**Figure 1.**
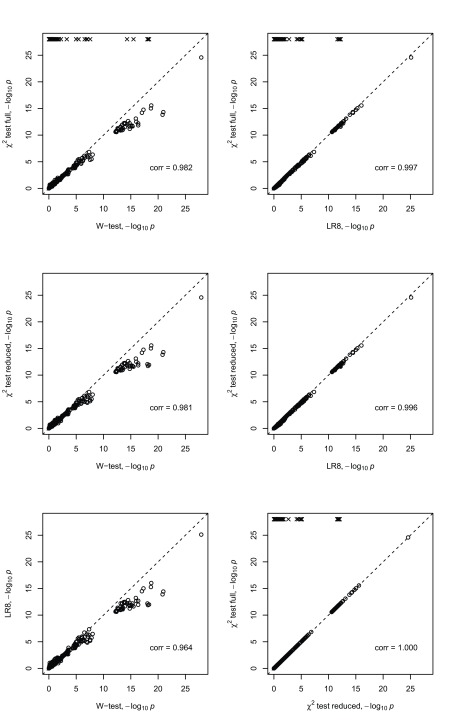
Scatter plots of negative log (base 10) transformed *P*-values from different interaction tests applied to the *W*-test demo data. The tests are the *W*-test, Pearson’s *χ* ^2^ test (full table), Pearson’s *χ* ^2^ test (reduced table), logistic regression with 8 df (LR8). The *W*-test demo data consists of 500 cases and 500 controls and 50 SNPs. The scatter plots show all 1225 SNP pair tests between the 50 SNPs. The squared Pearson product-moment correlation coefficient is shown in the bottom right of each plot. Crosses indicate points that did not evaluate due to empty cells in cases and/or controls.

**Figure 2.**
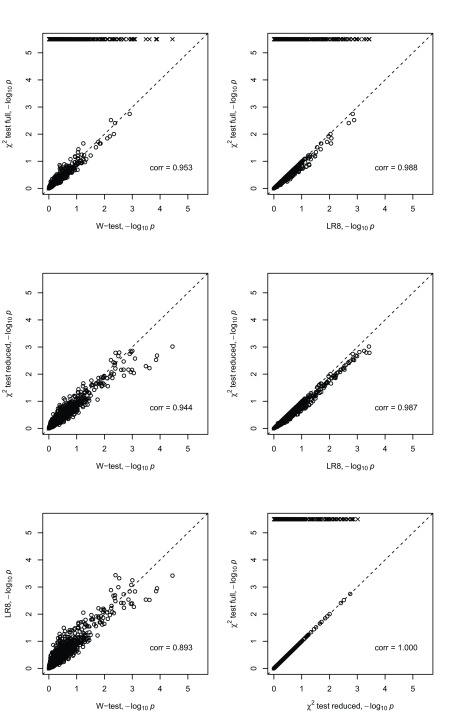
Scatter plots of negative log (base 10) transformed *P*-values from different interaction tests applied to the WTCCC2 data. The tests are the *W*-test, Pearson’s *χ* ^2^ test (full table), Pearson’s *χ* ^2^ test (reduced table), logistic regression with 8 df (LR8). The WTCCC2 data consists of a subset of 1000 female founders and 50 SNPs which were alternatively labelled as cases and controls. The scatter plots show all 1225 SNP pair tests between the 50 SNPs. The squared Pearson product-moment correlation coefficient is shown in the bottom right of each plot. Crosses indicate points that did not evaluate due to empty cells in cases and/or controls.

Figure 1 and Figure 2 show a comparison of the -log _10_ *P*-values achieved by the four different analysis methods in these two data sets. In each figure, comparisons of the *W*-test with other tests are shown in the left hand column, while comparisons between the various other tests are shown in the right hand column. For the *W*-test, we used the R software developed by Wang *et al.* ^1^ (with bootstrapping used to estimate parameters *h* and *f*); for Pearson’s *χ* ^2^ test and logistic regression we used the R functions chisq.test() and glm(), respectively. R version 3.3.3 was used throughout. The difference between the two *χ* ^2^ test implementations is that for one, denoted later as CHI-f, the chisq.test() function was applied to the full 2 *×*9 contingency table of counts of disease status versus genotype (which produces an 8 df test - provided there are no unobserved genotype categories) and for the other, denoted later as CHI-r, the chisq.test() function was applied to the vectors (each of length 1000) containing the individual-level phenotype and genotype data. With this latter implementation, the chisq.test() function automatically detects if there are unobserved genotype categories and removes them from consideration, resulting in a test with a potentially reduced number of degrees of freedom. Thus CHI-f corresponds to a *χ* ^2^ test for the ‘full’ genotype table, while CHI-r corresponds to a *χ* ^2^ test for a potentially ‘reduced’ genotype table.

We can see from Figure 1 and Figure 2 that the -log _10_ *P*-values achieved by the different methods are highly correlated and largely comparable in both data sets, except when Pearson’s *χ* ^2^ test fails to give a result (indicated in the plots by a cross and a -log _10_ *P*-value that we set arbitrarily to 4.5 or 28); this can occur with the the *χ* ^2^ full (CHI-f) implementation when one or more of the nine genotype categories does not appear. However, in these situations, the *W*-test, logistic regression and the reduced *χ* ^2^ (CHI-r) implementation all produce a result, and their results are seen to be largely comparable. The *W*-test does show slightly lower *P*-values at the most stringent significance thresholds when applied to the *W*-test demo data, suggesting a possible power advantage for the *W*-test over logistic regression and CHI-r for data generated under the simulation model assumed by Wang *et al.* The *W*-test also shows slightly lower *P*-values at the most stringent significance thresholds when applied to the WTCCC2 data. However, since these data were simulated under the null hypothesis of no association (or interaction), we cannot interpret this behaviour as implying higher power for the *W*-test.

### Application of *W*-test to simulated data sets

To further compare the performance of the *W*-test with previously proposed tests of association and/or interaction, we simulated 1000 replicates of genotype data for 500 cases and 500 controls at two diallelic loci with alleles denoted A and a (at locus 1) and B and b (at locus 2) respectively, under various generating models. For simulating under the null hypothesis, a higher number of replicates (5000) was used. Initially we assumed allele frequencies of (0.4, 0.6, 0.4, 0.6) for alleles (a, A, b, B) and assumed no linkage disequilibrium (LD) between the loci; these settings ensured that none of the tests were affected by issues of sparse data, so all tests could be computed in all simulation replicates.

For the *W*-test, the bootstrap procedure proposed by Wang *et al.* ^1^ is not possible when only two SNPs are being evaluated. We therefore considered two alternative approaches for specifying *h* and *f*. In the first (denoted as W), we assumed the test pair of SNPs had come from the real Wellcome Trust Case Control Consortium (WTCCC) ^12^ data set analysed by Wang *et al.* ^1^. This means that the estimates of *h* and *f* provided in the Supplementary Information (Supplementary Table S2) of Wang *et al.* ^1^ can be used. Given that Wang *et al.*’s ‘data-dependent’ estimation of *h* and *f* is derived primarily from the ‘other’ pairs of SNPs considered in the bootstrap procedure rather than from the pair of SNPs under test, we consider this a reasonable way to proceed, and, indeed, one could argue that these values of *h* and *f* should be suitable for use with any test pair of SNPs. Of course the number of individuals *N* in our current data set (500 cases and 500 controls) differs from the WTCCC data set, but, given that the bootstrap procedure only uses *N _B_* = min (1000, *N*) randomly chosen individuals, we would anticipate that this should not affect the results substantially. In the second approach (denoted as W′), we simply used the default values of *h* and *f* specified within the *W*-test software package (R version) i.e. *h* = ( *k −* 1) */k* and df *f* = *k −* 1 (where *k* is the number of genotype categories observed).

In addition to performing the *W*-test, Pearson’s *χ* ^2^ test (full and reduced versions) and logistic regression on 8 df (LR8), we also considered three additional logistic regression-based tests:

- LR3: logistic regression on 3 df, comparing (via a likelihood ratio test) the models logit( *p*) = *α* + *β x*_1_ + γ *x*_2_ + *i x*_1_ *x*_2_ and logit( *p*) = *α*
- LR1: logistic regression on 1 df, comparing (via a likelihood ratio test) the models logit( *p*) = *α* + *β x*_1_ + γ *x*_2_ + *i x*_1_ *x*_2_ and logit( *p*) = *α* + *β x*_1_ + *γx*_2_
- LRI: logistic regression on 1 df with only an interaction term, comparing (via a likelihood ratio test) the models logit( *p*) = *α* + *i x*_1_ *x*_2_ and logit( *p*) = *α*

These logistic regression-based tests all use an allelic (rather than a genotypic) coding of the genotype variables in order to reduce the df ^6, 8, 17^. This assumption of allelic effects has proved extremely effective in GWAS analysis of single SNPs ^12^ and is generally the default option used in most GWAS; even if the true effects do not precisely follow an allelic model, the reduction in df achieved can lead to higher power for tests that make this assumption ^18^.

While LR8, LR3 and LRI can be considered as tests of association allowing for interaction, (since the null hypothesis in each case corresponds to no relationship between genotype and phenotype), LR1 corresponds to a test of pure statistical interaction, under the assumption of an allelic model. Given the focus of interest of Wang *et al.* ^1^ on testing for epistasis, we additionally considered four other previously-proposed tests ^17^ designed to test pure statistical interaction: the Joint Effects (JE), Adjusted Wu (AWU), Adjusted Fast Epistasis (AFE) and Wellek and Ziegler (WZ) tests, all of which are implemented in our software package CASSI ( https://www.staff.ncl.ac.uk/richard.howey/cassi/downloads.html).

**Figure 3.**
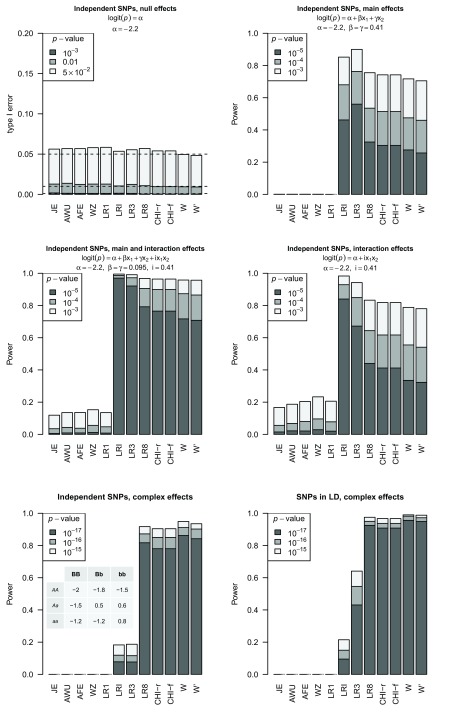
Power and Type I error plots for different effect models and tests. The simulating model is indicated above the plot for linear effects and for complex effects the log odds for each genotype combination is shown in the table in the bottom left plot. Tests considered are JE: Joint effects; AWU: Adjusted Wu; AFE: Adjusted Fast Epistasis; WZ: Welleck-Ziegler; LR1: Logistic regression with 1 df testing for interaction accounting for main effects; LR3: Logistic regression with 3 df testing for interaction and main effects; LR8: Logistic regression with 8 df testing for interaction and main effects, one parameter for every genotype combination between the two SNPs; LRI: Logistic regression with 1 df testing for interaction without accounting for main effects; CHI-f: full table *χ* ^2^ test with cell counts for every genotype combination between the two SNPs; CHI-r: reduced table *χ* ^2^ test where unobserved genotype categories are removed from consideration W: *W*-test with values of *h* and *f* estimated in Wang *et al.*’s Supplementary Table S2 using real WTCCC data; W *′*: *W*-test with default values of *h* and *f*.

Figure 3 shows the performance (type I error and power) of these tests under a variety of generating models. Given that no simulation replicates resulted in issues of sparse data, the performances of the full and reduced versions of Pearson’s *χ* ^2^ test (CHI-f and CHI-r) are identical, as expected. All tests show appropriate type I error rates under the null hypothesis of no association between genotype and phenotype (top left panel). In the presence of main effects only, the tests of pure interaction show appropriate type I error rates, whereas the tests of association allowing for interaction show vastly inflated error rates if considered as tests of interaction *per se* (top right panel, noting that ‘Power’ corresponds to power if considered as a test of the null hypothesis of no association, but corresponds to type I error if considered purely as a test of the interaction effect). Under the alternative hypotheses considered here, the tests of association allowing for interaction ( *W*-test, Pearson’s *χ* ^2^ test, LR8, LR3, LRI) show higher power than the tests of pure interaction (JE, AWU, AFE, WZ, LR1), consistent with the known lower power of interaction tests compared to tests of main effects ^19^.

The overall power of the *W*-test is seen to be similar to that of LR8 and Pearson’s *χ* ^2^ test; in some cases (e.g. Figure 3, bottom panels) the power of the *W*-test is higher than that of LR8 and Pearson’s *χ* ^2^ test, whereas in other cases (e.g. Figure 3, middle and top right panels) the power of the *W*-test is lower than that of LR8 and Pearson’s *χ* ^2^ test. The *W*-test with values of *h* and *f* estimated from genome-wide (WTCCC) data (denoted W) gave consistently slightly higher power than using the default values (denoted W′). For simulations under complex effects, where the generating model is not well-approximated by an allelic model ( Figure 3, bottom panels), the three genotypic tests ( *W*-test, *χ* ^2^ test, LR8) of association, allowing for interaction, all have higher power than the corresponding allelic tests (LR3, LRI), with the *W*-test (with values of *h* and *f* estimated from genome-wide data) showing the overall highest power. However, for simulations where the structure of the generating model matches an allelic model, ( Figure 3, middle and top right panels), the allelic tests show higher power than the genotypic tests.

Similar results to those described above were found when we repeated our simulations under further scenarios where the two loci were assumed to be in LD (data not visualised). We note that neither our simulations nor those presented by Wang *et al.* can be considered a fully comprehensive evaluation; given the effectively infinite number of possible association models that could exist between two SNPs and a disease phenotype, such an evaluation would be quite hard to achieve. It is thus very difficult to know in practice, in any given situation, which method is likely to have the highest power, making it difficult to specify in advance which test to use. The simulation results presented here are not intended to address the question of *how often* the underlying association model will be more or less amenable to testing using the *W*-test compared to alternative methods, but rather to point out that *there are situations* where existing alternative approaches show higher power. Our study thus redresses the balance in comparison to the study of Wang *et al.*, who only presented results from models where the *W*-test showed highest power.

In particular, in their simulation study, Wang *et al.* found the *W*-test to have considerably higher power than both Pearson’s *χ* ^2^ test and logistic regression (by which we believe they mean LR3, although they do not actually specify which logistic regression model they employed), particularly for low MAFs, see Figures 1 and 2 and Tables 1 and 2 of Wang *et al.* ^1^. The higher power of the *W*-test compared to LR3 is easily explained by the fact that the generating models used by Wang *et al.* did not follow an allelic pattern, which is the pattern that LR3 is best-configured to detect. The higher power compared to Pearson’s *χ* ^2^ test is more complicated to explain, but may be related to the fact that, in Wang *et al.*’s simulations, in addition to showing lower power, the *χ* ^2^ test also showed considerably lower *type I error rates* than the *W*-test (see Tables 1 and 2 of Wang *et al.*), with the *W*-test showing approximately correct (nominal) type I error rates and the *χ* ^2^ test showing *deflated* levels of type I error. While the correct control of type I error is an attractive property of the *W*-test, it makes the ‘powers’ shown by Wang *et al.* difficult to compare, since one would normally only compare power for tests that are well-calibrated, i.e. that show the same (correct) type I error rate.

**Figure 4.**
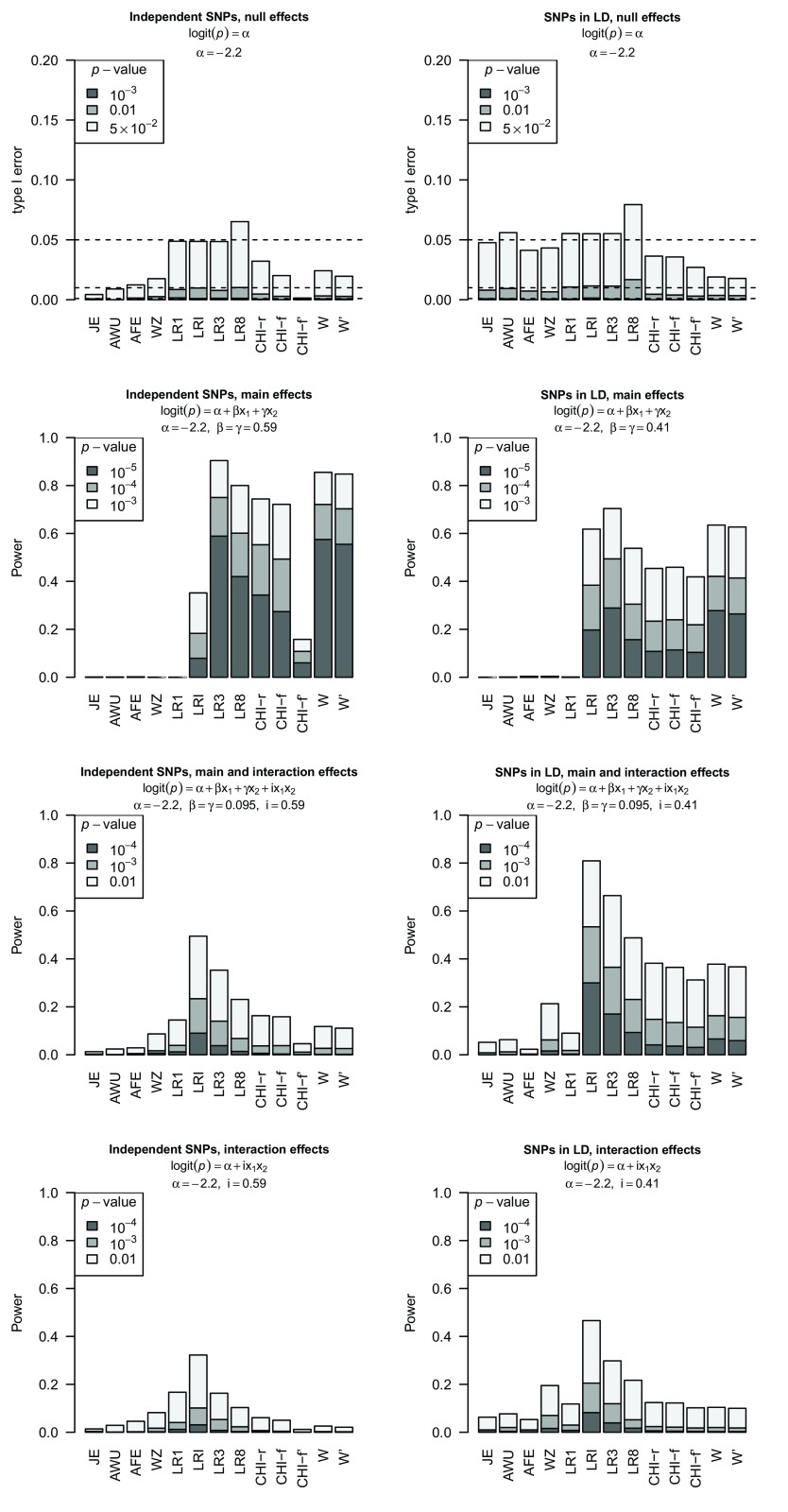
Power plots for different effect models and tests for SNPs with low minor allele frequencies (MAF=0.1). The simulating model is indicated above the plot. Test abbreviations are described in the legend to Figure 3 and CHI-f *′* is the *χ* ^2^ test where a undefined test result is counted as a non-detection and included in the denominator. Plots on the left show results for independent SNPs and plots on the right for SNPs in LD ( *R* ^2^ = 0.24 in controls, *R* ^2^ = 0.29 in combined cases and controls).

Given that this power difference was most profound at low MAFs, we wondered if it could be explained by the fact that, at low MAFs, we expect many *χ* ^2^ tests to be undefined (as demonstrated in Figure 1 and Figure 2) if implemented based on the full genotype table (CHI-f implementation). If one uses the CHI-f implementation and counts an undefined result (an ‘NA’) as a non-detection, while using the total number of simulation replicates (detections and non-detections) as the denominator, then this will result in a decrease in both power and type I error. We illustrate this phenomenon in Figure 4. Here we repeated our simulations using lower MAFs of 0.1 for the two SNPs and considered a variety of different scenarios both with and without LD. We denote by CHI-f *′* the power obtained when you count an undefined result as a non-detection, while using the total number of simulation replicates as the denominator. We denote by CHI-f the power obtained when you instead ignore undefined results, and use the number of detections as the numerator with the number of simulation replicates in which a result was obtained as the denominator. Figure 4 demonstrates that the CHIf *′* method of counting does indeed result in an apparent lower power for Pearson’s *χ* ^2^ test, although it is unclear whether this effect is sufficient to explain the differences in power presented by Wang *et al.* The powers of the *W*-test and LR8 remain, by and large, comparable, with sometimes one and sometimes the other showing highest power. However LR8 suffers from a slightly inflated type I error (whereas the *W*-test has an over-conservative type I error rate), suggesting that the *W*-test should be the preferred method in this instance. The allelic tests (LR1, LRI, LR3) all show well-calibrated type I error, while LRI and/or LR3 also show the highest power (particularly in the bottom four panels of Figure 4), reflecting the fact that the generating model in these simulations follows an allelic structure.

**Figure 5.**
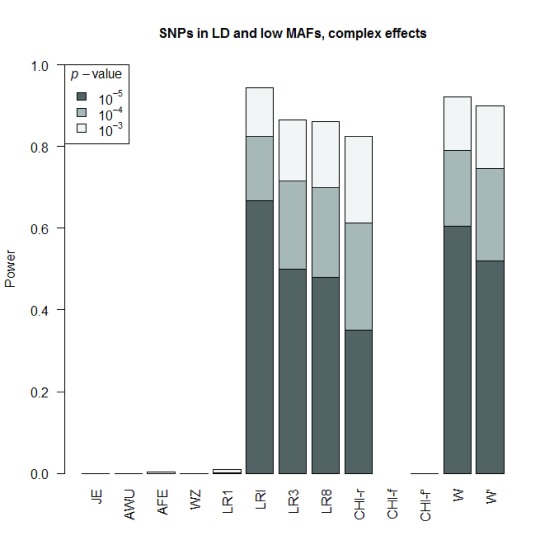
Power plots for SNPs showing complex effects with very low minor allele frequencies (MAF=0.01) and in strong LD ( *R* ^2^ = 0.64 in controls, *R* ^2^ = 0.83 in combined cases and controls). Test abbreviations are described in the legends to Figure 3 and Figure 4.

As a final comparison of methods under a situation that the *W*-test is specifically designed to address, we performed an additional set of simulations for pairs of SNPs with very low MAFs (MAF *≈*0.01 in controls, MAF *≈*0.03 in cases), in strong LD ( *R* ^2^ = 0.64 in controls, *R* ^2^ = 0.83 in combined cases and controls) and operating via complex effects (see generating model shown in the bottom left panel of Figure 3). The results are shown in Figure 5. In this instance, we can see that Pearson’s *χ* ^2^ test based on the full genotype table while ignoring undefined results (CHI-f) is undefined (0/0 replicates gave a significant result), while counting an undefined result as a non-detection (CHI-f *′*) has very low power (0/1000 replicates gave a significant result), possibly explaining its poor performance in Wang *et al.*’s simulations. The highest power is obtained with the logistic regression-based test LRI and with the *W*-test (with values of *h* and *f* estimated using real WTCCC data); reasonable but lower levels of power are seen with logistic regression-based tests LR3 and LR8, and with Pearson’s *χ* ^2^ test based on the reduced genotype table (CHI-r).

### Evaluation of previous application of *W*-test to bipolar disorder data sets

Wang *et al.* ^1^ also applied their *W*-test to two real genome-wide association data sets of bipolar disorder obtained from the Wellcome Trust Case Control Consortium (WTCCC) ^12^ and Genetic Association Network (GAIN). We show in Table 2 the SNP and gene pairs reported by Wang *et al.* as significant in WTCCC that they considered to be replicated in GAIN (see listing given in Wang *et al.*’s Supplementary Table S6 "Replicated and significant gene-gene interactions”), together with their WTCCC *W*-test *P*-values. Examination of the single-SNP *P*-values listed in Table 2 (and comparison of these *P*-values to those given in the original WTCCC publication ^12^) sounds an immediate warning note: these *P*-values seem suspiciously small for a modestly-sized GWAS of a complex neuropsychiatric disorder, and, more importantly, do not seem compatible with the results presented in Figure 4, Table 3 and Supplementary Table 7 of the original WTCCC publication (in which only one SNP, rs420259, achieved a *P*-value less than 5E-07 for bipolar disorder). Supplementary Figure S5 of Wang *et al.* (upper panel) shows the Manhattan plot obtained when using the single-SNP (main effects) version of Wang *et al.*’s *W*-test; this Manhattan plot does not seem consistent with the results presented in the original WTCCC publication. Even given that the most significant *P*-values have been cut out of Wang *et al.*’s Supplementary Figure S5 (owing to the choice of an upper limit of 10 for the *y* axis), the large number of remaining isolated significant SNPs, not supported by other SNPs in the same genetic region in LD with the significant SNP, suggests that these significant single-SNP results are highly likely to be artefacts, most likely due to genotyping errors.

**Table 2.** SNP pairs in WTCCC (with WTCCC *W*-test *P*-values) considered as replicated in GAIN.

			SNP Pair	SNP1 *^a^*	SNP2 *^a^*		
Network	SNP1 *^a^*	SNP2 *^a^*	*P*-value	*P*-value	*P*-value	Gene 1	Gene 2
1	rs6741692	**rs1048194**	5.84E-38	8.48E-06	**7.19E-25**	*DPP10*	*CENPN*
1	**rs3867492**	rs17108944	1.03E-27	**1.32E-23**	5.08E-03	*TMEM132D*	*NRXN3*
1	rs1864952	**rs1048194**	1.91E-35	2.93E-03	**7.19E-25**	*SLIT3*	*CENPN*
1	rs17637311	**rs3867492**	1.01E-25	1.38E-03	**1.32E-23**	*SLIT3*	*TMEM132D*
1	rs2407594	**rs1048194**	9.76E-36	5.49E-03	**7.19E-25**	*CSMD1*	*CENPN*
1	rs17068332	**rs3867492**	8.17E-28	6.50E-03	**1.32E-23**	*CSMD1*	*TMEM132D*
1	**rs3867492**	rs6030385	5.05E-24	**1.32E-23**	4.89E-03	*TMEM132D*	*PTPRT*
2	**rs17170832**	rs17135053	3.86E-18	**3.02E-06**	3.97E-03	*ELMO1*	*A2BP1*
2	**rs2849605**	rs17135053	3.28E-29	**4.27E-10**	3.97E-03	*PARK2*	*A2BP1*
2	**rs7666328**	rs9559408	4.82E-17	**1.90E-11**	2.75E-04	*NDST4*	*MYO16*
2	**rs7666328**	rs17135053	6.97E-16	**1.90E-11**	3.97E-03	*NDST4*	*RBFOX1*
2	**rs7666328**	rs1494451	1.31E-21	**1.90E-11**	1.60E-06	*NDST4*	*CNTNAP2*
2	**rs7666328**	**rs17170832**	9.99E-24	**1.90E-11**	**3.02E-06**	*NDST4*	*ELMO1*
2	**rs17170832**	rs13433234	3.08E-23	**3.02E-06**	3.68E-03	*ELMO1*	*MACROD2*
2	**rs11222695**	rs6043524	5.51E-11	**8.91E-03**	3.49E-05	*HNT*	*MACROD2*
2	**rs1494451**	rs13433234	7.43E-12	**1.60E-06**	3.68E-03	*CNTNAP2*	*MACROD2*
2	**rs2849605**	rs2785061	9.79E-19	**4.27E-10**	7.43E-03	*PARK2*	*ACCN1*
2	**rs2849605**	**rs11222695**	2.65E-25	**4.27E-10**	**8.91E-03**	*PARK2*	*HNT*

*^a^* SNPs marked in **bold** were flagged as unreliable by the WTCCC

Together with raw genotype data, the WTCCC distributes a list of SNPs ( exclusion-list-snps-26_04_2007.txt) that have failed various quality control checks and should therefore be considered unreliable. We compared the SNPs listed in Table 2 with those appearing in exclusion-list-snps-26_04_2007.txt and found that all but three of the rows of Table 2 contain at least one SNP that appears on the WTCCC recommended exclusion list. An additional list of 561 SNPs that failed manual visual checking of the cluster (intensity) plots was obtained from the WTCCC (Jeffrey Barrett, personal communication; Supplementary File 1). This list includes rs1048194, which appears in each of the remaining three rows of Table 2. Thus, every SNP pair identified by Wang *et al.* as significant in a pairwise *W*-test (of association allowing for interaction) in the WTCCC data contains at least one SNP that has been flagged as unreliable by the WTCCC. We therefore consider the WTCCC results presented by Wang *et al.* as highly suspect and likely to be explained by poor quality genotyping in cases, controls or both. We note that it is not necessary to obtain such lists of SNPs failing quality control from the WTCCC in order to spot this problem; visual inspection of the Manhattan plots (and comparison to the original WTCCC publication) is enough to at least flag up the problem, and implementation of standard GWAS quality control procedures would, in any case, eliminate the vast majority of these suspect SNPs.

Turning to the GAIN results presented by Wang *et al.*, we note that the single-SNP (main effects) Manhattan plot presented in Wang *et al.’*s Supplementary Figure S5 (lower panel) contains one highly significant result on chromosome 22. This result appears to correspond to an isolated significant SNP, not supported by other SNPs in the same genetic region in LD with the significant SNP, suggesting again that this result is likely to be an artefact attributable to genotyping error. In Table 3 we list the SNP and gene pairs in GAIN that Wang *et al.* consider to replicate their WTCCC findings (see listing given in Wang *et al.*’s Supplementary Table S6 "Replicated and significant gene-gene interactions”). We note that every row, but one, of the GAIN results contains SNP_A-8429018, which has a (suspiciously small) single-SNP *P*-value of 5.91E-18. We also note that SNP_A-8429018 (in Affymetrix nomenclature) corresponds to rs9606296 on chromosome 22, and thus most likely corresponds to the aforementioned likely artefactual result shown in Wang *et al.*’s Supplementary Figure S5 (lower panel).

**Table 3.** SNP pairs in GAIN (with GAIN *W*-test *P*-values) considered to replicate findings from WTCCC.

			SNP Pair	SNP1 *a*	SNP2 *a*		
Network	SNP1 *a*	SNP2 *a*	*P*-value	*P*-value	*P*-value	Gene 1	Gene 2
1	**SNP_A-8429018**	SNP_A-8715766	1.95E-23	**5.91E-18**	7.50E-03	*RTN4R*	*CSMD1*
1	SNP_A-2229791	**SNP_A-8429018**	1.18E-19	3.71E-03	**5.91E-18**	*SLIT3*	*RTN4R*
1	**SNP_A-8429018**	SNP_A-8630842	1.56E-22	**5.91E-18**	5.94E-03	*RTN4R*	*TMEM132D*
1	SNP_A-2050329	SNP_A-8715766	5.70E-11	0.001258	0.007499	*PTPRT*	*CSMD1*
1	**SNP_A-8429018**	SNP_A-8705647	1.11E-18	**5.91E-18**	4.56E-04	*RTN4R*	*DPP10*
2	**SNP_A-8429018**	SNP_A-8528492	1.68E-22	**5.91E-18**	5.36E-03	*RTN4R*	*MYO16*
2	SNP_A-2095851	**SNP_A-8429018**	3.18E-22	6.97E-04	**5.91E-18**	*ELMO1*	*RTN4R*
2	SNP_A-4233559	**SNP_A-8429018**	5.12E-20	2.28E-03	**5.91E-18**	*HNT*	*RTN4R*
2	SNP_A-2248688	**SNP_A-8429018**	4.69E-19	6.82E-03	**5.91E-18**	*PARK2*	*RTN4R*
2	**SNP_A-8429018**	SNP_A-8624018	1.87E-18	**5.91E-18**	4.87E-03	*RTN4R*	*ACCN1*
2	**SNP_A-8429018**	SNP_A-8599143	1.70E-19	**5.91E-18**	4.81E-03	*RTN4R*	*CNTNAP2*
2	SNP_A-8366063	**SNP_A-8429018**	5.12E-20	3.82E-03	**5.91E-18**	*RBFOX1*	*RTN4R*

*^a^* SNPs marked in **bold** appear unreliable based on the Manhattan plot presented by Wang *et al*.

The remaining row of GAIN results (see Table 3) contains SNP_A-2050329 and SNP_A-8715766, corresponding to rs3787282 and rs17070836 on chromosomes 20 and 8, respectively. The pairwise *W*-test *P*-value is given as 5.70E-11, with single-SNP *P*-values of 0.00125821 and 0.00749938. Although we have no particular reason to distrust this result, the fact that it is being interpreted as a replication of an interaction between *PTPRT* and *CSMD1* (genes which appear in our list of untrustworthy ‘significant’ WTCCC results shown in Table 2), means that, at best, we would consider this as an isolated finding requiring further replication.

**Figure 6.**
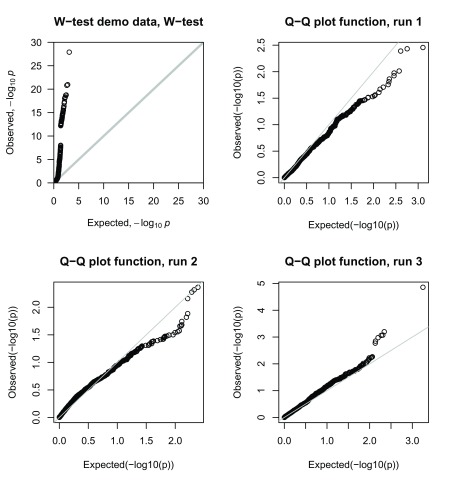
Q-Q plots of interaction tests. The top left plot shows a Q-Q plot of the *W*-test *P*-values using the *W*-test demo data. The remaining plots show Q-Q plots generated using the Q-Q plot function from the *W*-test R package each time using the same *W*-test demo data.

Figure 3 of Wang *et al.* ^1^ presents a quantile-quantile (Q-Q) plot of their pairwise *W*-test results from the real genome-wide data sets, although Wang *et al.* do not specify whether this Q-Q plot relates to results from WTCCC, GAIN or both. Unusually, this plot does not show any particularly significant results (no *P*-values less than 1.0E-07), even though, in their Supplementary Table S6, Wang *et al.* had listed a number of SNP pairs exceeding this level of significance. The legend of Wang *et al.*’s Figure 3 states that the *W*-test was computed on real genome-wide data with ‘permuted phenotype’. This seems an odd procedure; Q-Q plots are generally plotted using the real observed results (based on the real phenotypes) in order to determine whether the genome-wide distribution of test statistics is as expected ^12^. By definition, if one permutes the phenotype (to mimic data generated under the null hypothesis), one would indeed expect the Q-Q plot to follow the line of equality, but it does not provide any information about whether the observed results follow their expected distribution (and may thus be considered reliable). On closer investigation of the *W*-test software (R version) developed by Wang *et al.* ^1^, we found that the Q-Q plot function takes as input genotype data, but *not* phenotype data, and does indeed calculate *W*-test results using randomly permuted phenotypes with an equal number of cases and controls. Therefore, the function does not actually plot a Q-Q plot of the real observed results. As an example, Figure 6 shows the *W*-test demo data plotted as a standard Q-Q plot and then plotted three times using the Q-Q plot function from the *W*-test (R package) software. In contrast to the true *W*-test demo data results (which are highly significant, presumably because of the simulation model chosen by Wang *et al.*), the Q-Q plots generated within the *W*-test software suggest (somewhat misleadingly) that the observed test statistics are largely consistent with the null hypothesis of no association.

## Discussion

Here we have re-examined the novel *W*-test for pairwise epistasis recently proposed by Wang *et al.* ^1^ via theoretical evaluation, computer simulations and application to real data. We find that the *W*-test has strong similarities to both Pearson’s *χ* ^2^ test and logistic regression on 8 df, with the advantage over these tests (at lower minor allele frequencies) of generating no undefined test results, having no inflation in type I error, and in some cases – dependent on the underlying disease model assumed – showing higher power. For common variants, the advantages of the *W*-test over existing methods are less clear, and, indeed, there are situations where existing methods perform better.

In a real-data application, Wang *et al.* apply their *W*-test to genome-wide association data for bipolar disorder and highlight a number of significant detections of pairwise epistasis. We have not ourselves re-analysed these data using alternative methods, but, given the high level of similarity between the *W*-test and alternative methods found in analysis of real ( Figure 1 and Figure 2) and simulated ( Figure 3– Figure 5) genotype data, we anticipate that similarly significant results would be obtained when applying alternative methods such as logistic regression to the bipolar data. Unfortunately, all but one of the results presented by Wang *et al.* can most likely be attributed to SNP genotyping error (resulting in highly significant single-SNP *P*-values for one or both SNPs of a pair) and so are probably artefactual. We therefore consider the subsequent network analysis performed by Wang *et al.* of the identified interactions as being, at best, uninterpretable and, at worst, potentially highly misleading, and we urge researchers to exercise caution in their interpretation of these results. This warning illustrates the importance of using standard quality control checks (such as Q-Q and Manhattan plots of real observed test statistics) when analysing genome-wide association data, even when the primary focus of a study is on presenting novel methodology.

In summary, our investigations of the *W*-test suggest that this test remains an attractive option for testing for association, while allowing for interaction (although it does *not* test for pure interaction - the test can be sensitive to main effects of any or all SNPs included in the model), and it does offer some advantages over alternative tests (such as Pearson’s *χ* ^2^ test and logistic regression on 8 df) at lower minor allele frequencies. The fact that in some scenarios the *W*-test shows higher power, whereas in other scenarios alternative approaches show higher power, makes it difficult to specify in advance which test should be preferred. One attraction of logistic regression is its flexibility, allowing one to tailor the test to impose greater structure on the assumed alternative hypothesis (such as assuming multiplicative allelic effects), which can offer a power advantage when the imposed structure matches the true underlying data structure. Extensions of the *W*-test that would allow similar flexibility might be an interesting topic for further investigation.

## Data availability

The data referenced by this article are under copyright with the following copyright statement: Copyright: © 2017 Howey R and Cordell HJ

The *W*-test demo data set is distributed with the *W*-test software (R version) developed by Wang *et al.* and is available at: http://www2.ccrb.cuhk.edu.hk/wtest/download.html. The WTCCC data set contains the exclusion-list-snps-26_04_2007.txt file. This can be applied for at: https://www.ebi.ac.uk/ega/studies/EGAS00000000001. The WTCCC2 data can be applied for at: https://www.ebi.ac.uk/ega/studies/EGAS00000000028.

## Supplementary material

Supplementary File 1: List of 561 SNPs that failed manual visual checking of the cluster (intensity) plots performed by the WTCCC.
