# Supplementary material for: Further investigations of the W-test for pairwise epistasis testing
Source: Wellcome Open Res. 2017 Jul 21;2:54. [Version 1] doi: 10.12688/wellcomeopenres.11926.1 (PMC5553086; doi:10.12688/wellcomeopenres.11926.1)
Supplement: Peer review history

# Peer review: Referee response for version 1

**DOI:** 10.21956/wellcomeopenres.12891.r24398

**Published:** 2017-08-07

**Authors:** Wen-Hua Wei

**Affiliations:** 1 Department of Women's and Children's Health, Dunedin School of Medicine, University of Otago, Dunedin, New Zealand

Howey and Cordell have done an elegant job in assessing the W-test method recently published as a powerful approach for detecting pairwise epistatic interactions. Their comprehensive assessment covered the properties and caveats of the W-test, the key results of simulation and real data analysis in the original paper, as well as additional comparisons of W-test against a set of existing methods. The assessment led them to conclude that W-test had virtually no advantages over the existing methods in explicitly testing pairwise epistatic interactions, but could be a useful alternative in GWAS for detection of low frequency or rare variants.

 I like to thank the authors for their efforts because detection of epistasis or statistical gene-gene interaction has been challenging for a long time, and is longing for not only innovative but also robust methods and examples (Zietz H., *Am J Hum Genet* 100: 379-84, 2017). The study is well designed, well executed and technically sound. The paper is well written and easy to follow. I hope the comments below are helpful to improve the paper:

- Slightly surprisingly, neither the current nor the original W-test paper cited the recent review of detecting epistasis in human complex traits (Wei *et al.*, *Nat Rev Genet* 15: 722-33, 2014), where methods for testing interactions, rather than the whole pair effects, have been discussed. Issues of LD, marginal/main effects and capturing rare or low frequency variants have also been discussed in the review. Addition of the review in citation would make the discussion more interesting.
- It seems important to make clear that testing epistasis is to test interaction terms, not the overall effects of the whole pair. From that perspective, it might help to slightly reshape the presentation of the power results, e.g. high power in W-test when no interactions simulated to be interpreted as a disadvantage/problem instead? Nonetheless, it is good to point out that W-test can be a useful filter to select candidate pairs for explicit interaction tests, at a price of missing true interactions without important main effects.
- It seems also important to make clear that two markers in high LD carry little epistasis, although the pair could be statistically significant by haplotype effects. The property that a pair of closely located markers may capture rare haplotypes in the form of significant statistical epistasis has been explored for identifying rare variants and/or functional regulatory mechanisms (Wei *et al.* *PLoS One* 8: e71203, 2013; *Hum Mol Genet* 23: 5061-8, 2014). W-test seems to have advantages in this aspect, particularly in situations of multiple markers.

I have read this submission. I believe that I have an appropriate level of expertise to confirm that it is of an acceptable scientific standard.

## References

[1] WeiWHHemaniGHaleyCS: Detecting epistasis in human complex traits. *Nat Rev Genet*.2014;15(11) : 10.1038/nrg3747 722-33 10.1038/nrg3747 25200660

[2] WeiWGyeneseiASempleCAHaleyCS: Properties of local interactions and their potential value in complementing genome-wide association studies. *PLoS One*.2013;8(8) : 10.1371/journal.pone.0071203 e71203 10.1371/journal.pone.0071203 23940718PMC3733963

[3] WeiWHGuoYKindtASMerrimanTRSempleCAWangKHaleyCS: Abundant local interactions in the 4p16.1 region suggest functional mechanisms underlying SLC2A9 associations with human serum uric acid. *Hum Mol Genet*.2014;23(19) : 10.1093/hmg/ddu227 5061-8 10.1093/hmg/ddu227 24821702PMC4159153

# Peer review: Referee response for version 1

**DOI:** 10.21956/wellcomeopenres.12891.r24404

**Published:** 2017-08-04

**Authors:** Maggie Haitian Wang

**Affiliations:** 1 Division of Biostatistics and Centre for Clinical Research and Biostatistics, Chinese University of Hong Kong, Shatin, Hong Kong

In this paper, Howey and Cordell further investigated the theoretical properties of a novel W-test that measures pairwise epistasis effect, and applied the method in additional simulations. The authors especially compared the method to the logistic regression with 8 degrees of freedom. The simulation studies showed that the W-test has good/conservative type I error rates and better power than the Chi-squared test and Logistic regression under certain scenarios. Howey and Cordell pointed out a data quality control problem in the original paper, and raised concerned about the reported real bio-markers. The paper concluded that the W-test was a useful and practical method in real GWAS data analysis, especially for low frequency and rare variants.  The specific comments are as follows.

1. The authors raised an interesting point about the theoretical properties of the W-test. They argued that the Wang *et al *estimates the distribution of a pair of SNPs based on bootstrapping many other pairs, therefore pulling information from the entire data set. They suggestion that one could rather bootstrap only using a single pair to achieve a more accurate distribution. However, single-pair-based estimation would require that bootstrapping to be separately conducted for every pair under consideration, versus the current approach of Wang *et al. *that uses a pooled distribution from many pairs, such the B=200~400 bootstrapping is performed once for all. Single-pair based bootstrap falls in-between permutation tests and current approach. While Wang *et al’*s approach is more suitable for exhaustive pairwise evaluation in GWAS, the single-pair bootstrap is more suitable for candidate set study. A combination of the two methods could be realized in a stage-wise selection, the pooled distribution can be first used to screen the entire data set, followed by single pair bootstrap performed only for the top or significant interactions.
2. In the simulation study of the paper (Figure 5), the authors simulated a data with high LD and low frequency SNPs. When applying the W-test, the parameters h and f that determine the test distribution are borrowed from an external data with a different genetic architecture, rather than bootstrapping the working data. This would render the power of the W-test. One important advantage of the W-test is that the its distribution parameters are estimated using the working data to correct the bias of p-values due to complex data structures. Though in the current outcome (Figure 5), W-test still performed slightly better than LR8 and CHI-r, its performance should be even better if the distribution’s parameters are actually bootstrapped using the working data.  The same point also applies to other simulation results in Figure 3, 4, though the problem is less serious there as the simulation data are less complex.
3. We thank the authors to report the SNPs with genotyping errors and to share the list of exclusion SNPs. The real data analysis part by the W-test was re-done by us and posted on the following website: http://www2.ccrb.cuhk.edu.hk/wtest/materials/W-test%20-%20revised%204%20Aug%202017.pdf . This highlights the importance of data quality control, that inappropriate raw data processing would greatly affect the final result, and the importance of validation.
4. The Q-Q plot presented in Wang *et al. *aimed to show that the W-test’s estimated probability distribution under null hypothesis matches the data set’s distribution, therefore showing the reliability of the W-test p-values. The Q-Q plot was not intended to show the observed p-value distribution. The fact that the Q-Q plot was generated using “permutated phenotypes” was stated clearly in the original manuscript. However, the point of Howey and Cordell is well taken that QQ-plot is usually performed using observed data. The wtest R package has updated (in CRAN, and in http://www2.ccrb.cuhk.edu.hk/wtest/download.html ) to incorporate this option – user can choose to draw a QQ plot using either permutated phenotype or observed phenotype, according to different purposes.

I have read this submission. I believe that I have an appropriate level of expertise to confirm that it is of an acceptable scientific standard.
